# Self-locking Kirigami surfaces via controlled stretching

**DOI:** 10.1038/s44172-024-00169-5

**Published:** 2024-02-07

**Authors:** Qian Zhang, Ning Pan, Shuangbo Liu, Jian Feng, Jianguo Cai

**Affiliations:** Key Laboratory of C & PC Structures of Ministry of Education, National Prestress Engineering Research Center, Southeast University, Nanjing, 211189 China

**Keywords:** Engineering, Structural materials

## Abstract

Kirigami provides a powerful strategy to transform two-dimensional elements into complex three-dimensional functional structures with lengths ranging from nanoscale to microscale and macroscale. The stability and programmability of forming three-dimensional structures through mechanical actuation, whether external or self-balancing, are crucial. Here, we offer a system that performs the 2D to 3D transformation through sequential in-plane tension and release. As a result, the 3D state is obtained by out-plane popping and rotation and shows a self-locking behavior. The range of geometric parameters for kirigami elements with different stability properties is determined theoretically. The in-plane tension conditions are also calculated to break the transition point of the forming process. The horizontal and vertical modular array analysis demonstrates the scalability and programmability from the self-locking elements to the Kirigami surfaces. We expect that the kirigami pattern and design approach will serve for innovative systems, including tunable antennas, flexible electronics, and medical devices.

## Abstract (web summary)

Zhang, Pan, Liu, and their colleagues propose a self-locking kirigami pattern that utilizes in-plane tension and release to fold 3D configurations. This method has been applied to construct a series of modular surfaces.

## Introduction

Kirigami and origami, traditional arts of paper cutting and folding, have widespread applications in areas such as robotics^1–3^, biomedical engineering^4–6^, electromechanical systems^7–9^, metamaterials^10–13^, space structure^14^ and architectural structures^15^ due to their powerful capability of transforming two-dimensional patterns into complex three-dimensional structures^16–18^. In contrast to origami structures, kirigami introduces cuts to release constraints between facets, thereby increasing the variability and variety of achieved design configurations^19,20^. The realization of a kirigami-inspired three-dimensional configuration depends on the forming method^21^, including self-folding of stimuli-responsive materials, self-balancing by assembling prestressed structures, and external actuation.

Self-folding occurs when the material properties change in response to variations in external environments, including temperature^22^, light^23^, electric field^24^, and magnetic field^25^. This technique is crucial for advancing the frontier of origami/kirigami systems at micro- and nanoscales, where there are difficulties in increasing the actuation forces and response speed. The self-balancing comes from the fabrication of the stress-free intermediate structures on the prestretched elastomeric^26–28^. Compressive stresses are applied to these bonding points between the substrate and intermediate structures upon release of the prestretch, causing buckling processes that result in a transition into a three-dimensional shape. The development from planar to diverse curved substrates gives the method a fascinating appeal^29^. However, getting rid of the substrate makes it difficult to maintain a three-dimensional structural configuration.

External actuation uses external forces to induce stretching, bending, and folding of the kirigami elements^30^. It will continue to be a simple yet effective technique for making 3D origami/ kirigami structures at various length scales. There are three possible scenarios for the kirigami structure following the release of the external actuation. The first of which is to return to the initial planar state, and there is only one stable configuration^31,32^. The second one is that the three-dimensional configuration can be maintained due to large deformations or buckling instability during the forming process^33–35^. However, it may be able to return to its initial planar state under external out-plane forces. There are two stable configurations but there is no self-locking. The third one is that the maintained three-dimensional configuration may remain self-locking under out-plane loading and resist external perturbations. There are two stable configurations and a self-locking phenomenon. All three results have clear application scenarios, but there is currently a lack of awareness of the third. Here, we report a rotation-forming self-locking (RFSL) kirigami pattern^36^ and an in-plane active stretch forming method to fill this gap. The forming method of self-folding and self-balancing is suitable for isolated kirigami elements and can also be extended to modular arrays, which is an outstanding advantage over external actuation methods. In contrast, there are barriers to extending the external actuation forming method from kirigami elements to modular arrays^35^. In this paper, we therefore also aim to investigate the scalability of the proposed in-plane tension forming method for horizontal and vertical kirigami arrays and further explore their programmable properties.

## Results and Discussion

### Element active forming

**Fig. 1:**
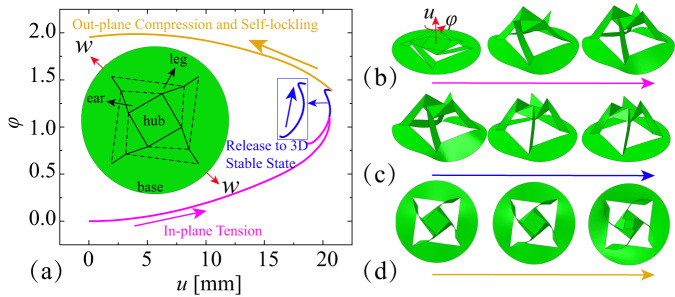
Active forming and self-locking behavior. **a** Configuration parameters (vertical upward displacement *u*, counterclockwise rotational angle *φ* and stretch displacement *w*) of the rotation forming self-locking (RFSL) kirigami element. **b**–**d** RFSL Kirigami element configurations for the three load scenarios: in-plane tension, release, and out-plane compression, respectively.

A rotationally symmetric kirigami pattern is designed with four L-shaped parts and a central square hub, as shown in Fig. 1. An L-shaped section consists of two plates, the ear and the leg. The central square hub, the ear plate, the leg plate and the circular base are connected by V-M-V (valley-mountain-valley) creases in turn. Moreover, the kirigami pattern is cut along the dashed lines shown in Fig. 1. There is no connection between adjacent parts. When the kirigami element is stretched along the axis of rotational symmetry, the central square hub moves vertically upwards and rotates counterclockwise simultaneously. The vertical and rotational displacements *u* and *φ* (element configuration parameters) correspond to the in-plane tension displacement *w* (element control parameter).

Figure 1a also illustrates the relationship between element configuration parameters of the kirigami element in three different external load scenarios. The corresponding simulation model information of the kirigami element is given in section Supplementary Note 1A and Supplementary Fig. 1, including geometry and material information, mesh and connection information, and loading regime. For the first load scenario, in-plane tension, the kirigami element is subjected to in-plane stretch along the rotational symmetry axis. Figure 1b shows the corresponding kirigami element configurations for different controlled stretches. As the controlled stretch increases, the vertical and rotational displacements increase first. The part of the base perpendicular to the stretch direction tends to deform downwards, forming a spherical shell. There is a critical stretch value, which corresponds to the motion mode transition point of the central square hub. After exceeding the critical value, the central hub starts the reverse motion, i.e., moves downwards and rotates clockwise. Corresponding stress distribution, vertical displacements and rotation angles of the kirigami elements are shown in Supplementary Fig. 2.

For the second load scenario, release to the stable 3D state, the external stretch is removed. The kirigami element deforms freely, guided by the strain energy gradient, as shown in Fig. 1c. The configuration parameters change slightly, but the controlled stretch and the strain energy are fully released. The central square hub appears to have an undulating movement, showing a complete process of up, down and up again. The rotation of the central square hub is slightly more pronounced, again alternating between counterclockwise and clockwise rotation. The release process produces a stable state that is different from the initial planar state, showing a transformation from a two-dimensional plane to a three-dimensional structure, also given in Supplementary Fig. 2. The active forming of the three-dimensional structure involves both in-plane tension and release processes.

The central square hub is applied with a vertical downward load for the out-plane compression scenario. Although the vertical displacement reduces gradually, the central square hub still rotates counterclockwise, as shown in Fig. 1d. The ear plates gradually fold closer to the central square hub along with the compression process. The kirigami element can not return to its initial planar state by means of out-plane compression. This is the self-locking behavior of the kirigami element, which reverts to its three-dimensional stable state even after the out-of-plane compressive load has been applied and released. This type of self-locking kirigami pattern, which has a rotational upward behavior during the active forming process, is recorded as the rotational forming self-locking (RFSL) kirigami element. The three-dimensional stable self-locking configuration can be achieved using particular in-plane controlled stretches and the corresponding release processes.

**Fig. 2:**
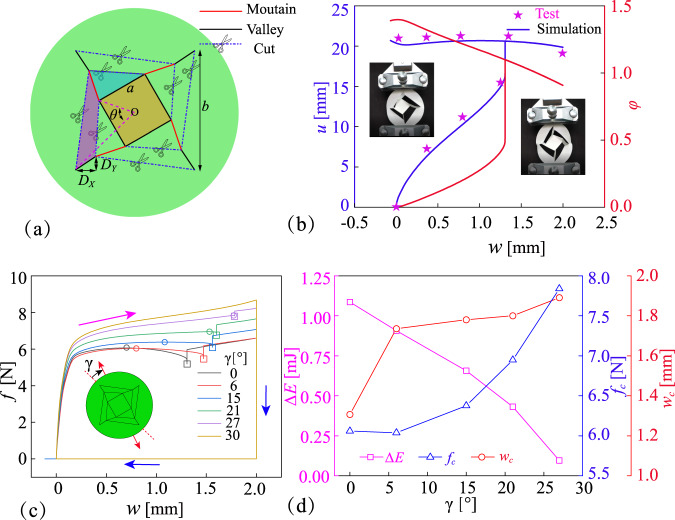
Transition of RFSL kirigami elements during the active forming. **a** Geometry parameters. **b** Experimental and simulation results on the configuration and control parameters (*u* and *φ* vs. *w*). **c** Influence of the stretching angle *γ* on the stretch load-displacement relationship. **d** Critical force *f*_c_, critical stretch *w*_c_ and strain energy gap Δ*E* for the transition points.

Five initial geometry parameters determine the RFSL kirigami pattern, as shown in Fig. 2a, including side lengths of the inner and outer squares *a* and *b*, the relative rotation angle *θ*, and the in-plane relative offset of vertex *D*_*X*_ and *D*_*Y*_. The mountain creases, valley creases and cut lines are assigned in the pattern. Physical models made of YUPO QJJ500 synthetic paper are used to carry out the controlled stretch test, detailed in Supplementary Figs. 3 and 4 and Supplementary Movie 1. Figure 2b compares the configuration parameters between the experimental and simulation results during the in-plane tension process. The errors in the vertical upward displacements between the experiment and the simulation are smaller than 5.0%. The experiment can give an overall picture of the morphological characteristics of the RFSL kirigami element during the active forming process. Both the vertical displacement and rotational angle of the central square hub increase sharply when the controlled stretch is close to the critical value *w*_c_ of 1.31 mm, corresponding to the transition point of the motion mode. For the stable three-dimensional state, there is a slight contraction of the kirigami element in the stretch direction, about −0.069 mm. At the moment, the vertical displacement of the central square hub can reach 20.7 mm, slightly larger than the side length of the inner square.

The stretch angle *γ* is defined as the offset angle between the stretch direction and the axis of rotational symmetry. Figure 2c represents the relationship between the stretch force *f* and stretch displacement *w* of the RFSL kirigami element with different stretch angles *γ*. When the stretch angle is less than 30°, the stretch load increases dramatically in the early stages (stretch displacement less than 0.15 mm) of the in-plane tensioning process. Thereafter, the load grows slowly to the peak *f*_c_, then decreases slightly. After a steep rise of stretch load in the stretch displacement of *w*_c_, the load gradually increases again with the stretch. The obvious rise in the force corresponds to the transition point of motion mode. At the moment, the strain energy of the kirigami element drops dramatically, recorded as the strain energy gap Δ*E*. So, it can be concluded that the critical load *f*_c_, and critical controlled stretch displacement *w*_c_ are the minimum stretch load and displacement required for the active forming of the RFSL kirigami element, respectively. When the stretch angle is 30°, there are only two phases of rapid and slow load growth in the in-plane tension processes. After the release process, the three-dimensional kirigami element returns completely to its initial two-dimensional planar state and does not shrink along the stretch direction. Active forming only can be achieved when the stretch angle is less than 30°, and the minimum stretch load and displacement are satisfied.

Figure 2d displays the critical force *f*_c_, critical stretch *w*_c_ and strain energy gap Δ*E* for the transition points of RFSL kirigami elements with various stretch angles *γ*. As the stretch angle gradually approaches 30°, the strain energy gap gradually decreases from 1.08 mJ to close to zero, while the required minimum stretch forces increase from 6.06 N to 7.84 N. The required minimum load and the minimum controlled stretch for active forming are extracted from different states, leading to the different relationship curves to the stretch angles. The controlled stretch displacement first increases rapidly from 1.31 mm to 1.74 mm, followed by a slight rise to 1.89 when the stretch angles are larger than 6°. The critical force and critical stretch can be regulated through configuration design and stretch design.

### Kirigami multistability

A geometric approach is proposed to approximate the morphology of the RFSL kirigami element based on the simplified virtual crease model during the in-plane tension process. As can be seen from Fig. 1 and Supplementary Fig. 2, the deformations of the central square hub and ear plates are much smaller than those of the leg plates during the active forming process. The central square hub and ear plates are assumed to be rigid plates, where the deformations are ignored. The deformation of the overall kirigami element is concentrated in the leg plates, and the geometric indicator *R* is established to characterize the bending and tearing deformations, which also can reflect strain energy information of the kirigami elements, as shown in Supplementary Fig. 5. The comparison of simulation and analytical results for the RFSL kirigami element in Supplementary Fig. 6 shows that the analytical method can accurately calculate the stable properties of the kirigami element.

**Fig. 3:**
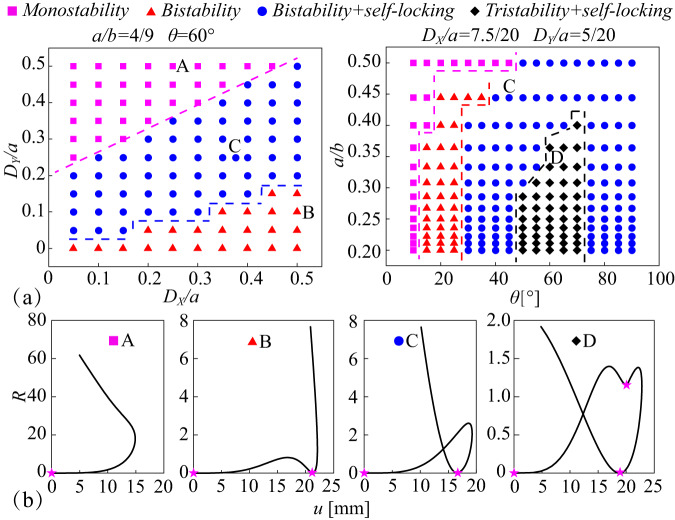
Kirigami Multistability. **a** Four kinds of kirigami elements, including monostability, bistability, bistability and self-locking combination, tristability and self-locking combination. **b** Relationship curves between the geometrical indicator *R* and vertical displacements *u* for the four types of kirigami element. The asterisk represents the stable state point.

Multistability is investigated for the kirigami elements with different geometry parameters, including monostability, bistability, bistability and self-locking combination, tristability and self-locking combination, as shown in Fig. 3. The influence of the geometry parameters on the multistability of kirigami elements is investigated in Fig. 3a. When the kirigami elements are designed with *a*/*b* = 4/9 and *θ* = 60°, only three modes of monostability, bistability, bistability and self-locking combination occur for various in-plane relative offsets of the vertex. The mode of bistability and self-locking combination tends to appear when the vertex offsets in both directions are close. When the offset *D*_*X*_ is smaller than the offset *D*_*Y*_, the kirigami elements are more likely to be monostable, and vice versa tend to be bistable. The corresponding relationship curves between the geometric indicator *R* of leg plates and vertical displacements *u* of center square hubs are also demonstrated in Fig. 3b. For all kirigami elements, the vertical displacements of central square hubs all show a tendency to increase to a peak and then decrease. For monostable kirigami elements, the geometrical indicator *R* always goes up. There is only one local point of minimal strain energy, corresponding to the initial two-dimensional planar state. For the bistable kirigami elements, another extreme point exists on the branch where the vertical displacement rises. But for this new stable configuration, the kirigami elements will revert from the three-dimensional states to their initial planar state when out-plane compression is applied to the central square hub. For the self-locking kirigami element with bistability, another stable state occurs on the branch where the vertical displacement falls. The kirigami elements discussed in Figs. 1 and 2 are of this type.

Another parametric analysis is carried out to discuss the influence of the side length ratio of the inner and outer squares *a*/*b*, the relative rotation angle *θ* for the kirigami elements with the predesigned relative vertex offsets *D*_*X*_*/a* = 7.5/20 and *D*_*Y*_*/a* = 5/20. When the ratio *a*/*b* of the kirigami element is not greater than 0.4, the four modes of monostability, bistability, bistability and self-locking combination, tristability and self-locking combination appear in sequence as the relative rotation angle increases. The modes of bistability, tristability and self-locking combination tend to disappear when the side length of the inner square gradually approaches half of the outer one. For the self-locking kirigami element with tristability, two other stable states are located on the vertical displacement ascending and descending branches, respectively. The stable state on the ascending branch is not self-locking, while the stable state on the descending branch has the self-locking property.

### Modular Kirigami surface

RFSL kirigami elements are assembled into two-dimensional modular surfaces. The active forming of three-dimensional surfaces by the in-plane tension or out-plane induction can offer a wide range of application prospects for the programmable kirigami surface. The vertical heights and rotation angles of the individual central square hubs can provide unique local morphological information, greatly enhancing the information space of modular kirigami surfaces.

#### In-plane rectangular array

**Fig. 4:**
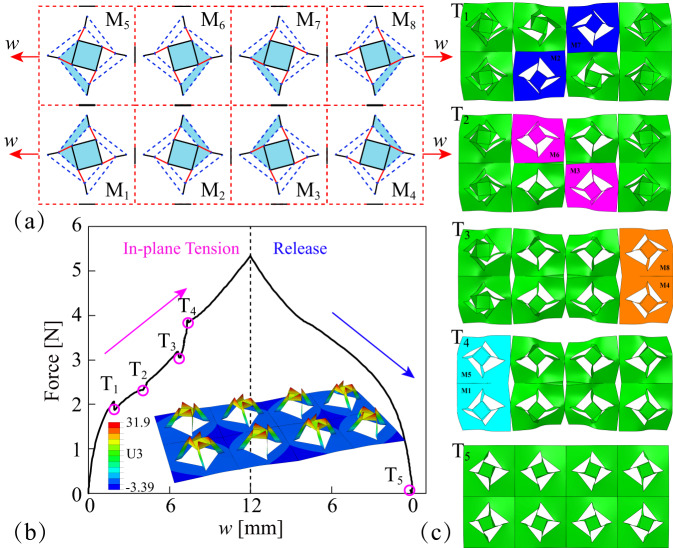
In-plane rectangular array of rotation forming self-locking (RFSL) kirigami elements. **a** Array design of eight symmetrical kirigami elements M with weak connections. **b** Active forming process of the modular kirigami surface, including in-plane tension and release. **c** Surface configurations corresponding to transition points T of kirigami elements.

An in-plane 2 × 4 rectangular array modular model is designed to illustrate the scalability of the RFSL kirigami element with geometry parameters: *a* = 20 mm, *b* = 45 mm, *θ* = −60°, *D*_*X*_ = 7.5 mm, *D*_*Y*_ = 5.0 mm, as shown in Fig. 4. The adjacent RFSL kirigami elements are arranged symmetrically and are partially connected on the symmetry axis. This weak connection ensures that the external tension between the connected kirigami elements can be transmitted, but also, the independence of the active forming of the RFSL kirigami element can be achieved by reducing the interaction between the connected elements. Figure 4a shows that the modular model is subjected to the in-plane horizontal stretch applied to the edges of the kirigami surface.

Figure 4b displays the measured tensile forces during the complete active forming process of the modular kirigami surface, including two scenarios, in-plane tension and release. It is immediately apparent from the diagram that in the in-plane tension process, the stretch force always follows an upward trend except for some fluctuation (T1, T2, T3 and T4) corresponding to the transition of RFSL kirigami elements, as shown in Fig. 4c. Supplementary Fig. 7a also represents the relationship curve between the configuration parameters and control parameters (*u* and *φ* vs. *w*) for M1, M2 and M6 kirigami elements. Before the T1 state, the central square hubs of all RFSL kirigami elements move upward, but the progress is not consistent. For the state T1, the RFSL kirigami elements M2 and M7 in the center of the modular surface complete the transition first and enter the local self-locking state. Next, the other two kirigami elements M3 and M6 in the center also finish the active forming in the state T2. At the states T3 and T4, the four RFSL kirigami elements in the edge achieve the transition in two sequential groups, M8 and M4, M5 and M1. The non-consistent active forming process of symmetrical kirigami elements demonstrates the designability of modular kirigami surfaces. Figure 4b and Supplementary Fig. 7b also reveal that the kirigami surface exhibits a uniform forming configuration in the state T5 after all the external loads of RFSL kirigami elements have been released. Furthermore, each RFSL kirigami element in the modular array also maintains its out-of-plane self-locking characteristics, as shown in Supplementary Fig. 7c. The in-plane active forming method provides prerequisites for the assembly of different kirigami elements and allows the design of three-dimensional kirigami surfaces with complex patterns by controlled stretches and predesigned patterns, such as the pattern shown in Supplementary Fig. 8. For the in-plane rectangular array of RFSL kirigami elements in Supplementary Fig. 8a, different maximum stretches will lead to different stable configurations. Supplementary Fig. 8b displays a two-dimensional planar kirigami surface consisting of many identical kirigami elements at inconsistent angles to the tensioning direction. After the in-plane tensioning and release process, parts of the kirigami elements can be shaped while others will revert to the planar state, resulting in the overall formation of the desired three-dimensional pattern.

#### Out-plane vertical array

Out-plane vertical multi-layer arrays are designed to obtain a high vertical height for a given in-plane dimensional limit. The design of a multi-layer array can be achieved by embedding a new layer of smaller size RFSL kirigami element on the central square hub of the former layer. All layers have been theoretically calculated, as shown in Fig. 3, to have bistability and self-locking characteristics in order to obtain the self-locking vertical array. The geometry parameters and pattern of the four-layer vertical array based on RFSL krigami elements are given in Supplementary Table 1 and Supplementary Fig. 9a.

**Fig. 5:**
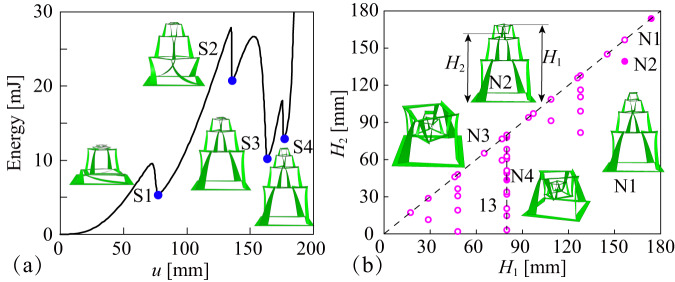
Out-plane vertical four layers array of rotation forming self-locking (RFSL) kirigami elements. **a** Relationship between the strain energy of the kirigami array and the vertical displacement *u* of the central square hub during the forming process. There are four transition states S1, S2, S3 and S4. **b** Programmability investigation of four layers array in terms of the relative relationship between the array vertical height *H*_1_ and the vertical position of the central square hub *H*_2_. Four configurations N1, N2, N3 and N4 are given as examples.

Figure 5a demonstrates the forming process of the four-layer vertical array, induced by the out-plane stretch loads on the central square hub. There are large fluctuations in the strain energy, and the four extreme points S1, S2, S3 and S4 correspond to the transition of EFSL kirigami elements, the second layer, the third layer, the first layer and the fourth layer, respectively. The forming process can also be reflected by the jump information in the rotation angle of each layer, as shown in Supplementary Fig. 9b. A jump in the relative rotation angle to the lower layer indicates the completion of forming the kirigami element in that layer. The order in which the layers of RFSL kirigami elements are formed reflects the relationship between the minimum required external loads of the different layers. Kirigami elements requiring larger external induced loads are formed later. Prior to the state S1, the RFSL kirigami element in each layer moves upward and the corresponding height of each layer increases. But the unfolding height of the second layer accounts for the major part. There is the highest strain energy at the state S2, mainly because the unformed layers have higher strain energy, especially the kirigami element of the first layer. At state S3, where the RFSL kirigami element of the first layer is formed, there is an obvious drop in the strain energy of the vertical array relative to state 2. An additional stable state point S4 exists nearby, with a slight increase in both strain energy and vertical displacement, corresponding to the formation of the fourth RFSL kirigami element with a minimum size. In addition, the assembled vertical four-layer array retains its out-plane self-locking properties, shown in Supplementary Fig. 9c. The array does not transform from its three-dimensional configuration to its initial two-dimensional planar configuration when the central square hub of the fourth layer is subjected to compressive loads.

The morphological programmability of this four-layer vertical array is further analyzed in Fig. 5b. The relative relationship between the array vertical height *H*_1_ and the vertical position of the central square hub *H*_2_ is systematically investigated. For the second, third and fourth layers of RFSL kirigami elements, there are generally two relative position relationships of the corresponding central square hub in relation to its base, with the central square hub in a higher and lower position, known as the “1” and “−1” state, respectively, as shown in Supplementary Fig. 10. And there is an unformed state known as the “0” state. The forming process of the four-layer vertical array in Fig. 5a can be thought of as a transition from the “0” “0” “0” “0” state to the “1” “1” “1” “1” state, which has the highest overall height with the corresponding layer heights of 79.91 mm, 47.95 mm, 28.77 mm and 17.26 mm respectively. At this state, two heights *H*_1_ and *H*_2_ are equal, both 173.89 mm. There are 39 different self-locking states of the four-layer vertical array when the central square hubs of all layers are constrained above the base of the first layer, and the detailed information is given in Supplementary Table 2. The height distribution of the vertical array is also given in Fig. 5b, supplemented by four examples to further illustrate the configurations of the four-layer vertical array of RFSL kirigami elements. Configuration N2 corresponds to the “1” “1” “1” “−1” state with heights *H*_1_ and *H*_2_ of 156.63 mm and 139.37 mm, respectively. It is worth noting that for configurations N3 and N4, although the overall heights *H*_1_ are the same at 79.91 mm, the heights of the central square hub *H*_2_ are different, being 77.99 mm (state “1” “−1” “1” “1”) and 43.47 mm (state “1” “−1” “1” “1”) respectively, which provides an effective way of hiding the height information of central square hub using the overall height information. Moreover, there are up to 13 configurations of the four-layer vertical arrays with different central square hub heights *H*_2_, when the overall height is 79.91 mm. Similarly, the information on the rotation angles of the RFSL kirigami elements in each layer contributes to the information complexity of the vertical array.

## Methods

### Simulation model information

The kirigami element is designed with geometry parameters: side length of the outer square edge *a* = 20 mm; side length of inner square facet *b* = 45 mm; rotation angle of the inner square relative to the outer square (positive if counterclockwise) *θ* = −60°; in-plane relative offset of vertex of the triangle facet relative to the corresponding one of the outer square edge *D*_*X*_ = 7.5 mm and *D*_*Y*_ = 5.0 mm. The polyethylene terephthalate (PET) film is employed in the modeling with a mass density of 1380 kg/m^3^, elastic modulus of 3.2 GPa, Poisson’s ratio of 0.43, and thickness of 0.4 mm. S4R and S3R elements are selected to simulate the facets connected by hinges (CONN3D2) along the creases, including the valley creases and mountain creases, as shown in Supplementary Fig. 1a. The rotational stiffness of the crease is relatively small compared to the bending and tensile stiffness of the plates, so the rotational stiffness is regarded as zero in the simulation models. In the in-plane tension process of the analyzed kirigami element, the controlled stretches (*w* = 2.0 mm) are applied along with the diagonal direction of the out-square facet. The other five degrees of freedom of these two points are constrained, as shown in Supplementary Fig. 1b. It is noted that an initial vertical displacement of 0.1 mm is introduced prior to in-plane tension to induce the deformation direction and avoid the plane bifurcation behavior. Besides, the static general step is used to simulate the motion process. In the release process, the above-mentioned tensile loads are suppressed, and the model deforms itself to release the stored strain energy, as shown in Supplementary Fig. 1c. The constraint conditions are kept unchanged. In the out-plane compression process, the vertical compressive displacements *u* are applied to the central square hub of the deformed model, as shown in Supplementary Fig. 1d.

### Geometric approach for motion process

A geometric approach is proposed based on the simplified virtual crease model to approximate the morphology of the RFSL kirigami element during the in-plane tension process. The basic assumptions of the geometric approach can be summarized as follows. (1) There is no deformation in the central square hub and ear plates. (2) The ear plate rotates freely along the valley crease connected to the central square hub, with a crease angle of *β*, as shown in Supplementary Fig. 5. (3) There is no deformation in the base of the kirigami element, and the creases between the leg plates and the base are also hinged. (4) There is a virtual crease set along the diagonal line of each leg plate, such as the line AE on the leg plate AIEL. For the leg plate with assumed deformation, lengths of lines AI, IE, EL and LA do not change between the initial two-dimensional planar and stable three-dimensional forming states. (5) There will be changes in the virtual crease length and the distance between points *L* and *I* by comparing the three-dimensional stable state with the corresponding initial planar state. The geometric indicator *R* is introduced and defined as $R=\varDelta {L}_{LI}^{2}+\varDelta {L}_{AE}^{2}$. (6) The crease angle of *β* during the active forming process can be determined using the optimized method for minimum geometric indicator *R*.

A coordinate system is established with the out square facet as the reference, and point O is selected as the origin of the coordinate system. So, the coordinates of points A, E and I can be expressed as

1 $$\overrightarrow{OA}=\left(\frac{\sqrt{2}}{2}a\,\cos \left(-\frac{3}{4}{{{{{\rm{\pi }}}}}}+\theta \right),\frac{\sqrt{2}}{2}a\,\sin \left(-\frac{3}{4}{{{{{\rm{\pi }}}}}}+\theta \right),0\right)$$

2 $$\overrightarrow{OE}=\left(-\frac{b}{2},-\frac{b}{2},0\right)$$

3 $$OI=\overrightarrow{OE}+({D}_{X},{D}_{Y},0)$$

Points D and L can be obtained by rotating points A and I clockwise by 90° around the origin, respectively. The corresponding coordinates are given by

4 $$\overrightarrow{OD}=\overrightarrow{OA}\cdot {{{{{{\bf{T}}}}}}}^{{{{{{\rm{T}}}}}}},\overrightarrow{OL}=\overrightarrow{OI}\cdot {{{{{{\bf{T}}}}}}}^{{{{{{\rm{T}}}}}}}$$

where **T** is the transformation matrix, can be expressed as

5 $${{{{{\bf{T}}}}}}=\left[\begin{array}{ccc}\cos (-{{{{{\rm{\pi }}}}}}/2) & -\,\sin (-{{{{{\rm{\pi }}}}}}/2) & 0\\ \sin (-{{{{{\rm{\pi }}}}}}/2) & \cos (-{{{{{\rm{\pi }}}}}}/2) & 0\\ 0 & 0 & 1\end{array}\right]$$

During the active forming process, the central square hub moves upwards and rotates with displacements of *u* and *φ*, respectively. The coordinates of points A_1_ and D_1_ after motion corresponding to points A and D can be calculated by

6 $$\overrightarrow{OA1}=[\overrightarrow{OA}+(0,0,u)]\cdot {{{{{{\bf{T}}}}}}}_{\varphi }^{{{{{{\rm{T}}}}}}},\overrightarrow{OD1}=[\overrightarrow{OD}+(0,0,u)]\cdot {{{{{{\bf{T}}}}}}}_{\varphi }^{{{{{{\rm{T}}}}}}}$$

where **T**_*φ*_ is the transformation matrix corresponding to a counterclockwise rotation of *φ*, expressed as

7 $${{{{{{\bf{T}}}}}}}_{\varphi }=\left[\begin{array}{ccc}\cos \varphi & -\,\sin \varphi & 0\\ \sin \varphi & \cos \varphi & 0\\ 0 & 0 & 1\end{array}\right]$$

Substituting Eqs. (1), (3) and (6) into ${L}_{{{{{{\rm{AI}}}}}}}={L}_{A1{{{{{\rm{I}}}}}}}$ yields the constraint relationship between vertical displacement *u* and rotational angle *φ* of the central square hub. Besides, the coordinate of point L_2_ after motion corresponding to point L can be calculated by

8 $$\overrightarrow{OL}_{\!1} \,= 	\, [\overrightarrow{OL}+(0,0,u)]\cdot {{{{{{\bf{T}}}}}}}_{\varphi }^{{{{{{\rm{T}}}}}}},{\overrightarrow{{A}_{1}{L}_{1}}}=\overrightarrow{OL}_{1}-\overrightarrow{OA}_{1}\\ \overrightarrow{{A}_{1}{L}_{2}}\,= 	\, \overrightarrow{{A}_{1}{L}_{1}}\times \,\cos (-\beta )+\left(\frac{\overrightarrow{{D}_{1}{A}_{1}}}{|\overrightarrow{{D}_{1}{A}_{1}}|}\times \overrightarrow{{A}_{1}{L}_{1}}\right)\times \,\sin (-\beta )\\ 	+\left(\frac{\overrightarrow{{D}_{1}{A}_{1}}}{|\overrightarrow{{D}_{1}{A}_{1}}|}\times \left(\frac{\overrightarrow{{D}_{1}{A}_{1}}}{|\overrightarrow{{D}_{1}{A}_{1}}|}\cdot \overrightarrow{{A}_{1}{L}_{1}}\right)\times {\left(\right.}1-\,\cos (-\beta )\right)\overrightarrow{{OL}_{2}} \\ =	 \ \overrightarrow{{A}_{1}{L}_{2}}+\overrightarrow{{OA}_{1}}$$

where point L_1_ corresponds to point L when only the vertical and rotational displacements are considered, and at this moment, the crease angle *β* is regarded to be zero. Then, the geometric indicator *R* can be expressed as

9 $$\varDelta {L}_{AE} =	 \ |\overrightarrow{OE}-\overrightarrow{OA}|-|\overrightarrow{OE}-\overrightarrow{{OA}_{1}}|,\varDelta {L}_{LI} \\ =	\ |\overrightarrow{OI}-\overrightarrow{OL}|-|\overrightarrow{OI}-\overrightarrow{{OL}_{2}}|\\ R =	\ \varDelta {L}_{LI}^{2}+\varDelta {L}_{AE}^{2}$$

At any state during the motion, the solve problem of crease angle *β* can be transformed into an optimization problem with crease angle *β* as the variable and a geometric indicator as the optimization objective. Function *fmincon* in MATLAB is utilized to calculate the minimum geometric indicator.

### Element experiments

The experimental study was carried out to verify the active forming and self-locking behavior of RFSL kirigami elements. The physical model was made of YUPO QJJ500 synthetic paper with a thickness of 0.5 mm. Side lengths of the inner and outer squares *a* and *b* are 1125/19 mm and 500/19 mm, respectively. The corresponding relative rotation angle *θ* is −60°. The in-plane relative offset of vertex *D*_*X*_ and *D*_*Y*_ are 375/38 mm and 125/19 mm, respectively. Besides, the radius of the base is 50 mm. A universal testing machine was used for the test with displacement control mode. In our experimental model, we cut nicks in the plate to represent the crease. In other words, only a small thickness is retained to connect adjacent plates. The rotational stiffness of the crease is relatively small compared to the bending and tensile stiffness of the plates. The rotational stiffness is ignored. So in our simulation, no rotational stiffnesses are introduced for the mountain and valley creases.

## Conclusion

The active forming and self-locking behavior of RFSL elements are thoroughly investigated, and the assembly arrays of kirigami elements in planar and vertical directions are further discussed. We characterize the four multistability properties of general kirigami elements based on the simplified virtual crease model and uncover the corresponding geometry conditions, including the RFSL elements with bistability and self-locking. The simulation and experiment results show that RFSL elements can transform from the 2D planar state to the 3D stable state through in-plane tension and release processes. From the experiments and simulations, it can be found the morphology that we focus on and material properties and scale are not closely related. The results can also be verified through theoretical analysis. The analysis of out-plane popping and rotation behavior reveals the crucial transition points in the active forming process and further specifies the stretch range, minimum stretch forces and displacements required for active forming. It paves the road to program the 3D configuration design of the RFSL elements.

Modular kirigami surfaces assembled by the RFSL elements can achieve self-locking and programable configurations. The active forming process of the in-plane rectangular array demonstrates the feasibility of the in-plane stretch induction method for the RFSL element assembly. The forming sequences of kirigami elements and the active design assembly pattern can provide a great deal of information space for kirigami surfaces. Similar scalability and programmability analyses are also performed for the four-layer vertical array, as illustrated by the 39 and 13 combinations of central square hub heights for the vertical array with unrestricted and restricted overall heights, respectively.

The stable self-locking kirigami surface induced by in-plane tension holds great promise for applications in many fields, such as reconfigurable surfaces, high-precision sensors and flexible electronic systems. Electronic components lacking out-of-plane driving space or those requiring the maintenance of operational performance while subject to out-of-plane load disturbances can both benefit from its use. Furthermore, this self-locking performance of the kirigami element is comparable to that of spring components. To reflect the level and distribution of the out-of-plane load, it might be actively constructed as an out-of-plane load sensor. The usage of designed surfaces can provide good conformability and recognize the morphological characteristics of irregular surfaces, such as the existence of local protrusions.

### Supplementary information

Supplementary Information

Description of Additional Supplementary Files

Supplementary Video 1

## Data Availability

The data that support the plots within this paper and other findings of this study are available from the author upon reasonable request.

## Code availability

The codes that support the findings of this study are available from the corresponding author upon reasonable request.
