# Supplementary material for: Self-locking Kirigami surfaces via controlled stretching
Source: Commun Eng. 2024 Feb 7;3:26. doi: 10.1038/s44172-024-00169-5 (PMC10955878; doi:10.1038/s44172-024-00169-5)
Supplement: Supplementary file 1 — Supplementary Information [file 44172_2024_169_MOESM1_ESM.pdf]

Self-Locking Kirigami Surfaces via Controlled Stretching

Supplementary Materials

Qian Zhang, Ning Pan*, Shuangbo Liu*, Jian Feng, Jianguo Cai†.*

** These authors contributed equally to this work;*

† Corresponding author E-mail: j.cai@seu.edu.cn

Key Laboratory of C & PC Structures of Ministry of Education, National Prestress Engineering Research Center, Southeast University, Nanjing 211189, China

This file includes:

Supplementary Note 1. Element Active Forming Simulation 2
A. Simulation model 2
B. Simulation results 3
Supplementary Note 2. Element Experiments 4
A. Active forming 4
B. Self-locking 5
Supplementary Note 3. Element Active Forming Analytical Method 6
A. Geometric approach 6
B. Finite element simulation verification 7
Supplementary Note 4. Planar Array Analysis 8
A. Array elements 8
B. Pattern design 9
Supplementary Note 5. Vertical Array Analysis 10
A. Scalability 10
B. Programmability 11

Supplementary Figures 1 to 10

Supplementary Tables 1 to 2

Supplementary Note 1. Element Active Forming Simulation

A. Simulation model

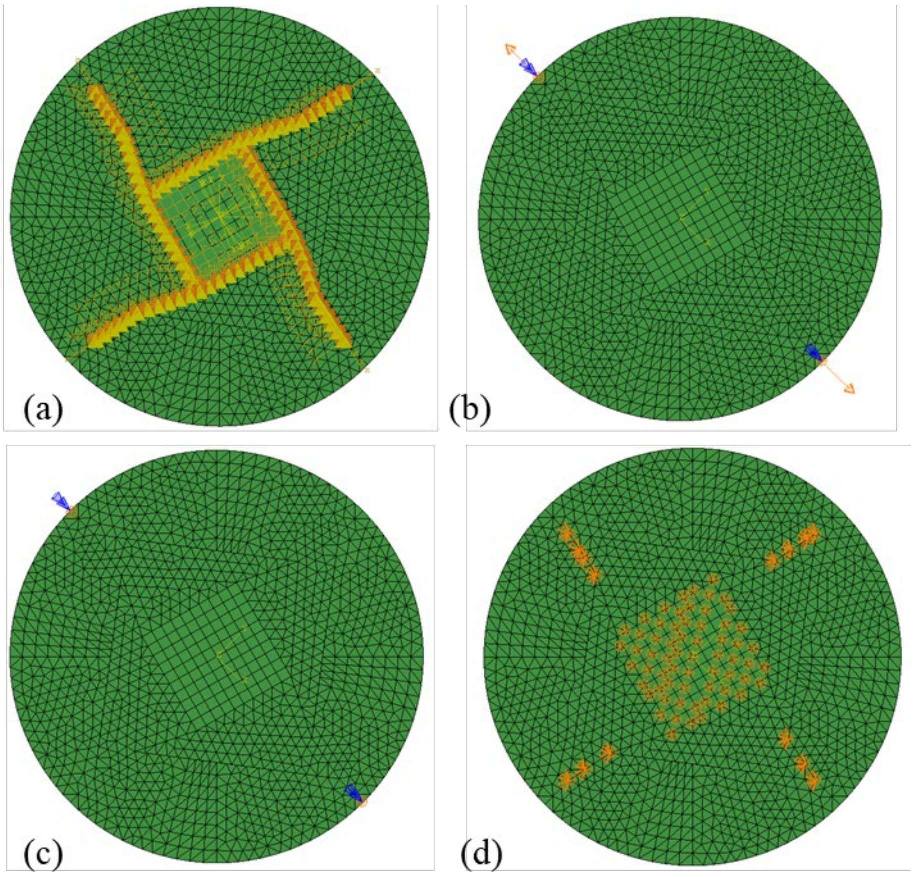

Supplementary Figure 1: Simulation model information. (a) Connections between the different facets. (b) Boundary conditions for the in-plane tension process. (c) Boundary conditions for the release process. (d) Boundary conditions for the out-plane compression process.

B. Simulation results

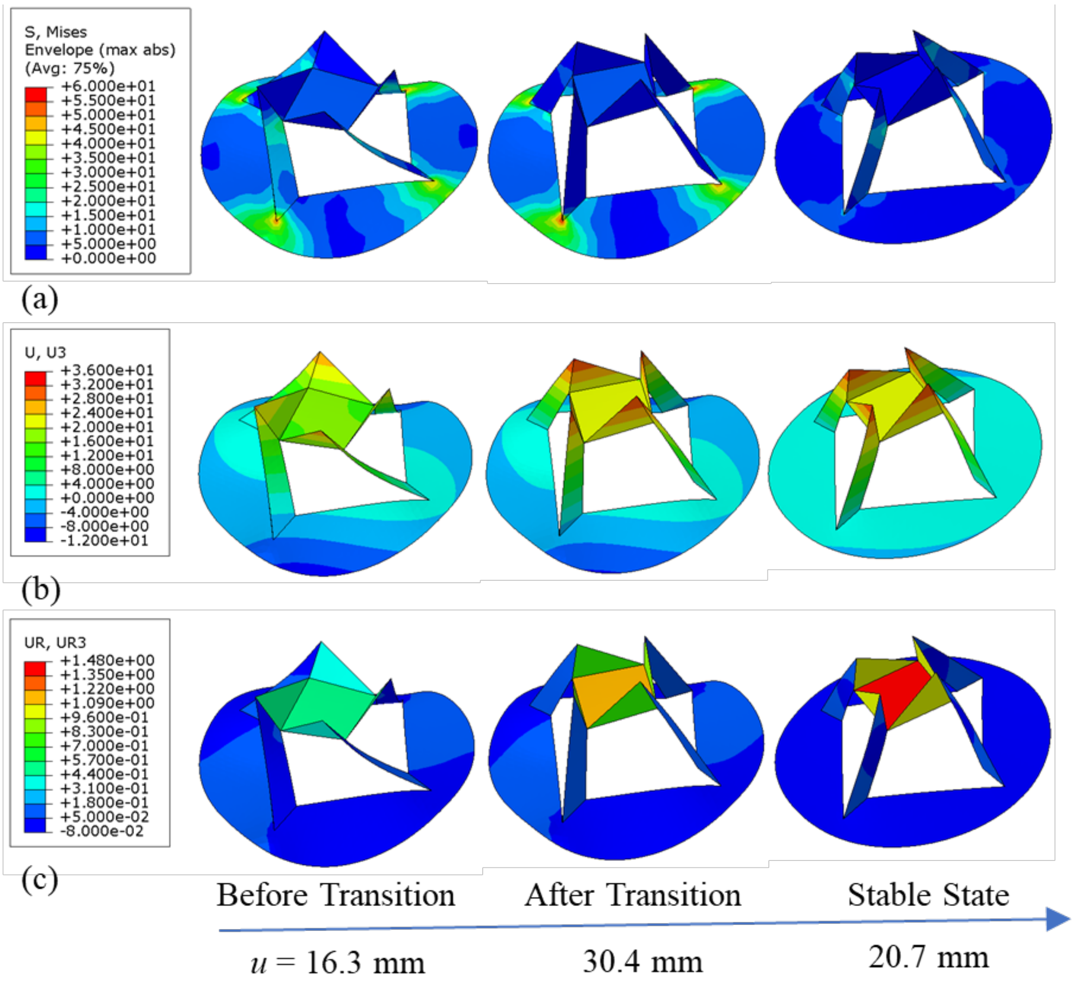

Supplementary Figure 2: Deformation information of the RFSL kirigami element in three states, before and after transition, stable state. (a) Stress (S, Mises MPa). (b) Vertical displacement (U3 mm). (c) Rotation angle (UR3). Before and after the transition point, the controlled stretches change slightly, and the structural stress distribution also varies very little, but the overall vertical and rotational displacements change dramatically.

Supplementary Note 2. Element Experiments

The physical model was made of YUPO QJJ500 synthetic paper. Our cutting machine failed to cut PET, making it difficult to achieve precise cutting. After multiple damage to the props, we attempted to replace them with multi-layer paper (YUPO QJJ500 synthetic paper). The experimental results obtained from different types of paper are basically consistent for the morphology, and consistent experimental phenomena can be observed. By using different material parameters for simulation analysis, consistent results for the morphology can also be obtained, although the forces are inconsistent. So, we believe that the morphology that we focus on and material properties are not closely related.

A. Active forming

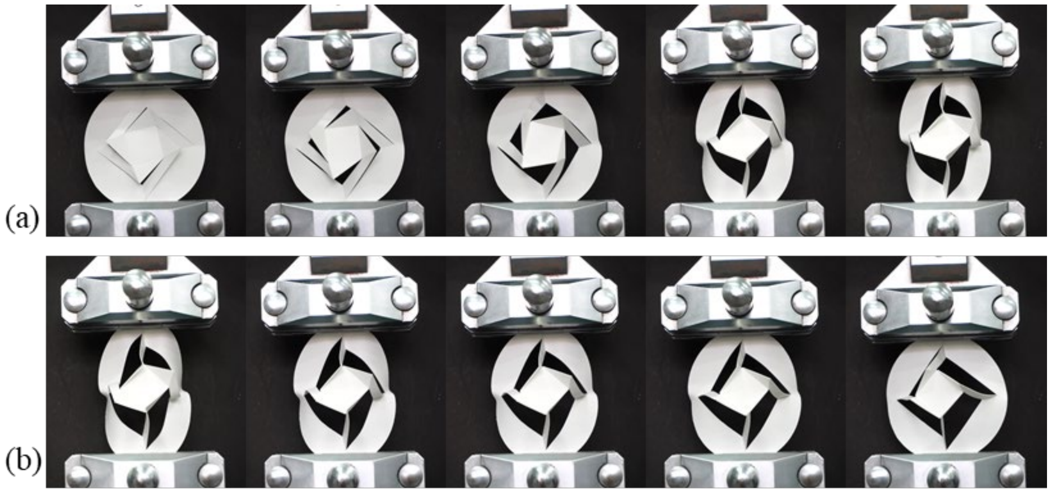

Supplementary Figure 3: Active forming process of the RFSL kirigami element. (a) In-plane tension. The paper model was clamped diagonally at both ends. A controlled stretch of $w = 11.0$ mm was applied to the upper end. The central square hub moved upwards and rotated. (b) Release. The applied stretch was gradually released, and the corresponding base deformation decreased. The central square hub moved downwards and rotated in reverse, which reveals that active forming has been achieved.

B. Self-locking

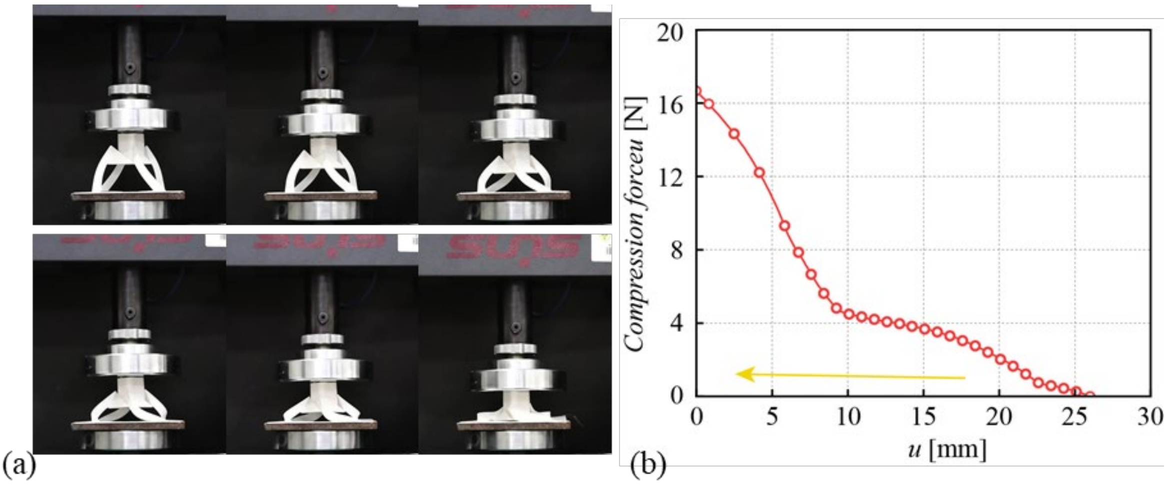

Supplementary Figure 4: Self-locking behavior of the RFSL kirigami element. (a) Out-plane compression. From the forming state of the RFSL kirigami elements, a compression displacement of 26.0 mm was applied to the central square hub. The foam column was designed to transfer compressive loads, where the top and bottom were glued to the upper loading end of the universal testing machine and the central square hub of the paper model, respectively. The base of the RFSL kirigami element was placed freely on the platform of the testing machine, allowing for rotation. (b) Compressive loads during the out-plane compression process. The compression load always increased, and the rate of increase of the compression load became greater in the later stages.

Supplementary Note 3. Element Active Forming Analytical Method

A. Geometric approach

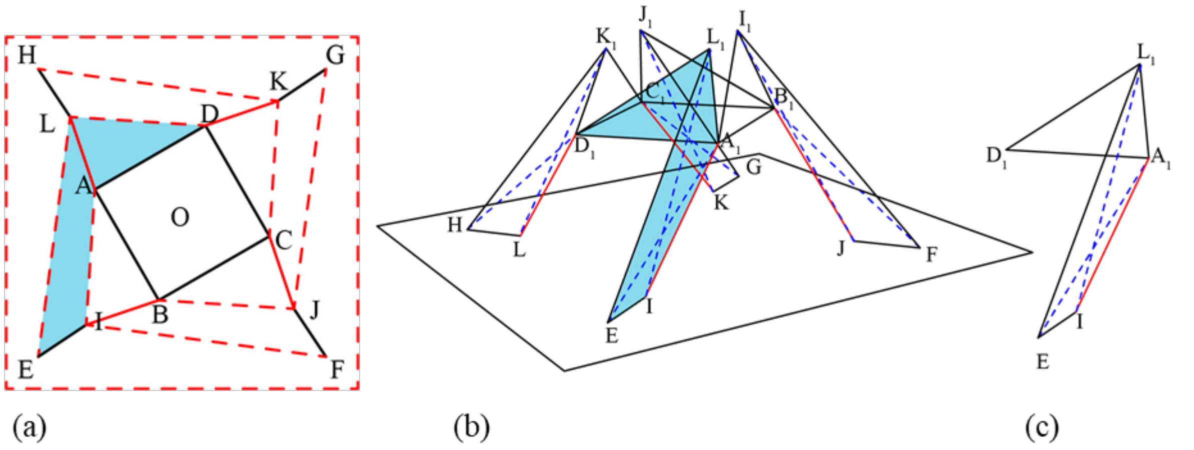

Supplementary Figure 5: Simplified virtual crease model of the RFSL kirigami element. (a) Initial planar state. (b) Stable three-dimensional state after active forming. (c) One L-shaped part with assumed concentrated deformation.

B. Finite element simulation verification

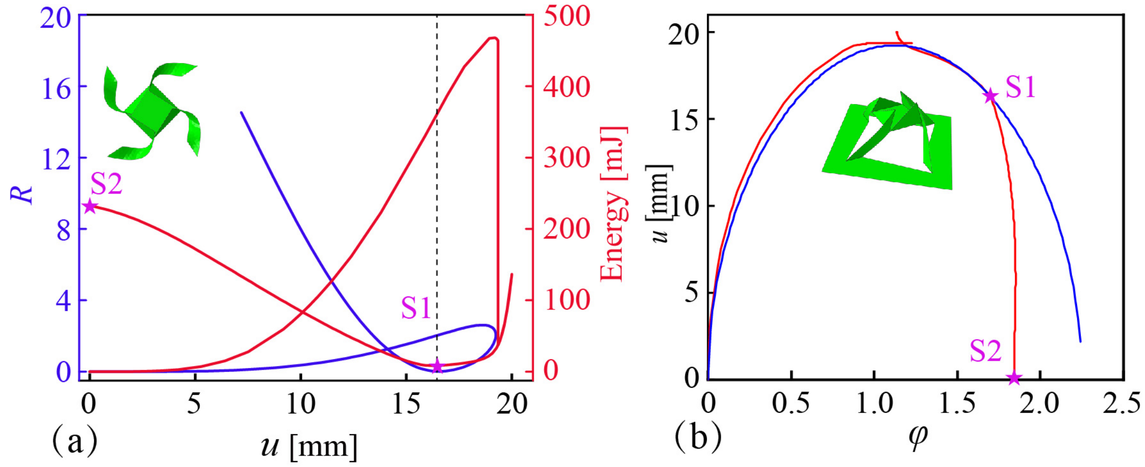

Supplementary Figure 6: Comparison of simulation and analytical results for the RFSL kirigami element with geometry parameters: a = 20 mm, b = 45 mm, θ = -60°, D_X = 7.5 mm, D_Y = 5.0 mm. (a) Strain energy and geometric indicator R. In the finite element simulation analysis, the maximum vertical displacement is 20.0 mm. The geometric indicator R can reflect the changing trend of strain energy. The positions of the stable state S1 obtained by the two methods are the same, and both methods can indicate that the kirigami element has the self-locking property. (b) Relationship between the rotation angle φ and the vertical displacement u. The results show the configuration parameters of the RFSL kirigami element near the stable states can be obtained accurately. However, for the larger deformations (e.g., S2), the assumptions of the geometric approach deviate from the real element deformation, leading the poor calculation results.

Supplementary Note 4. Planar Array Analysis

A. Array elements

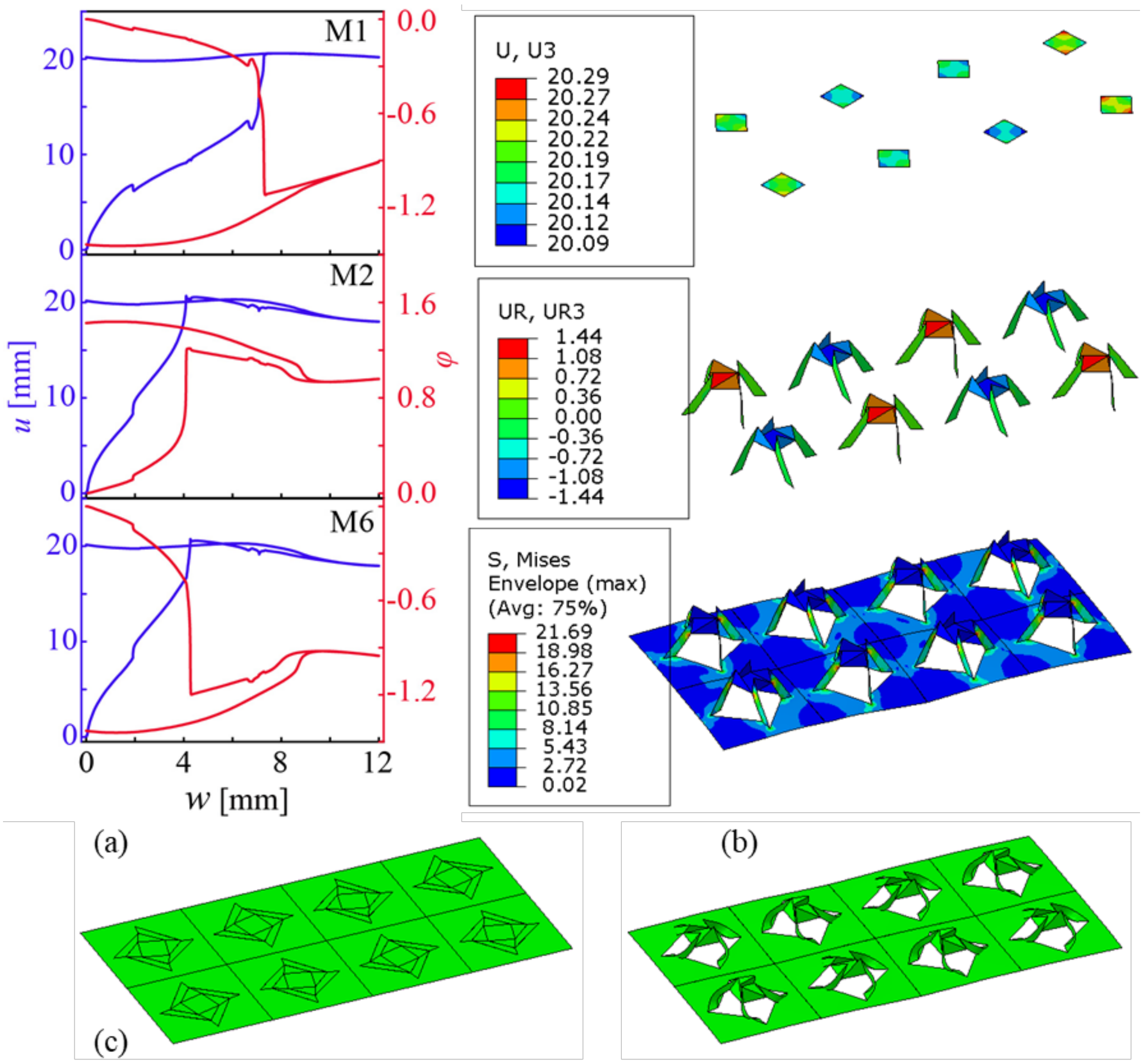

Supplementary Figure 7: Array element analysis. There are eight identical RFSL kirigami elements designed with geometry parameters: $a = 20$ mm, $b = 45$ mm, $\theta = -60°$, $D_X = 7.5$ mm, $D_Y = 5.0$ mm in the square base with sides of 80.0 mm. And horizontal stretches w of 12.0 mm were applied at the midpoint of both ends. (a) Relationship between the configuration parameters and control parameters (u and φ vs. w) for M1, M2 and M6 kirigami elements. (b) Element Information of the modular kirigami surface, including the vertical displacements of the central square hubs, rotational angles of kirigami elements, and stress of modular kirigami surface. (c) Initial planar state and the self-locking state.

B. Pattern design

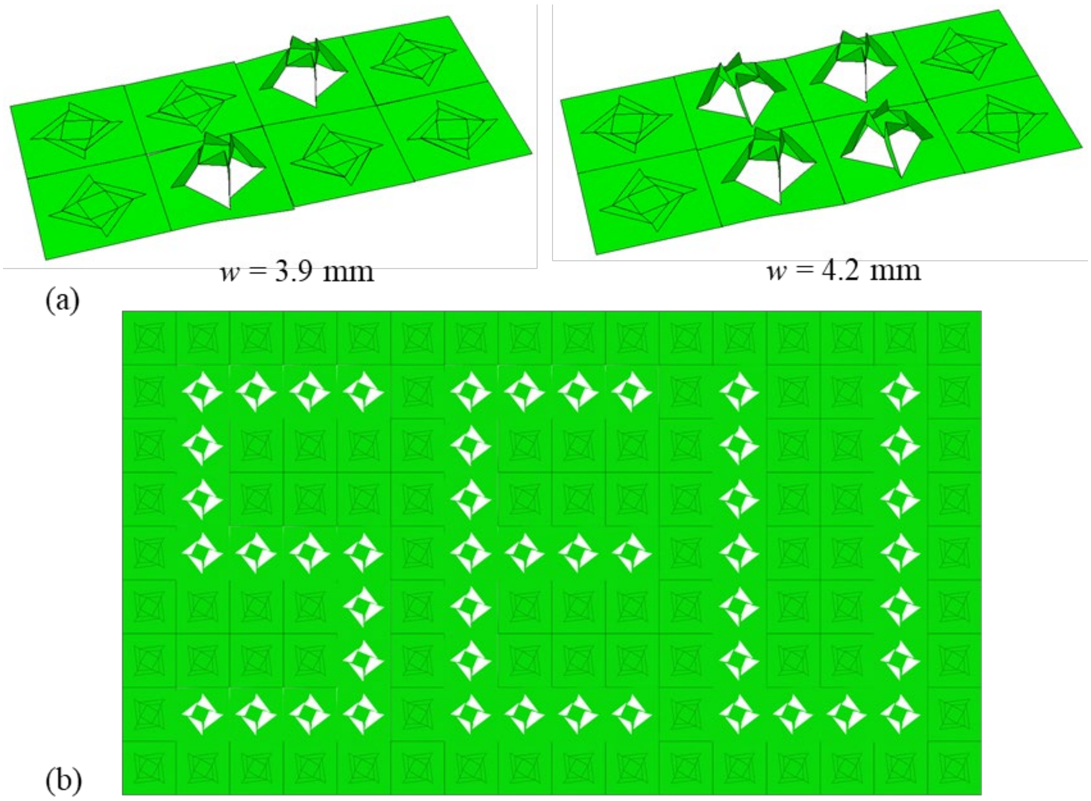

Supplementary Figure 8: Example of formable patterns. (a) Controlled stretch. For the in-plane rectangular array of RFSL kirigami elements, different maximum stretches will lead to different stable configurations. (b) Pre-designed patterns. A two-dimensional planar kirigami surface consists of many identical kirigami elements. The surface is similarly stretched in the planar direction, while the kirigami elements are at inconsistent angles to the tensioning direction according to the pre-design. After the in-plane tensioning and release process, parts of the kirigami elements can be shaped while others will revert to the planar state, resulting in the overall formation of the desired three-dimensional pattern, such as "SEU", the abbreviation of " Southeastern University". The programmability of the kirigami elements and the corresponding assembly method greatly expands the design space for 3D patterns.

Supplementary Note 5. Vertical Array Analysis

A. Scalability

Supplementary Table 1. Geometry parameters of a four-layer vertical array of RFSL krigami elements.

Layers	a [mm]	b [mm]	θ [°]	D_X [mm]	D_Y [mm]
First layer	1000/9	700/9	80	175/9	50/9
Second layer	200/3	140/3	80	35/3	10/3
Third layer	40.0	28.0	80	7.0	2.0
Fourth layer	24.0	16.8	80	4.2	1.2

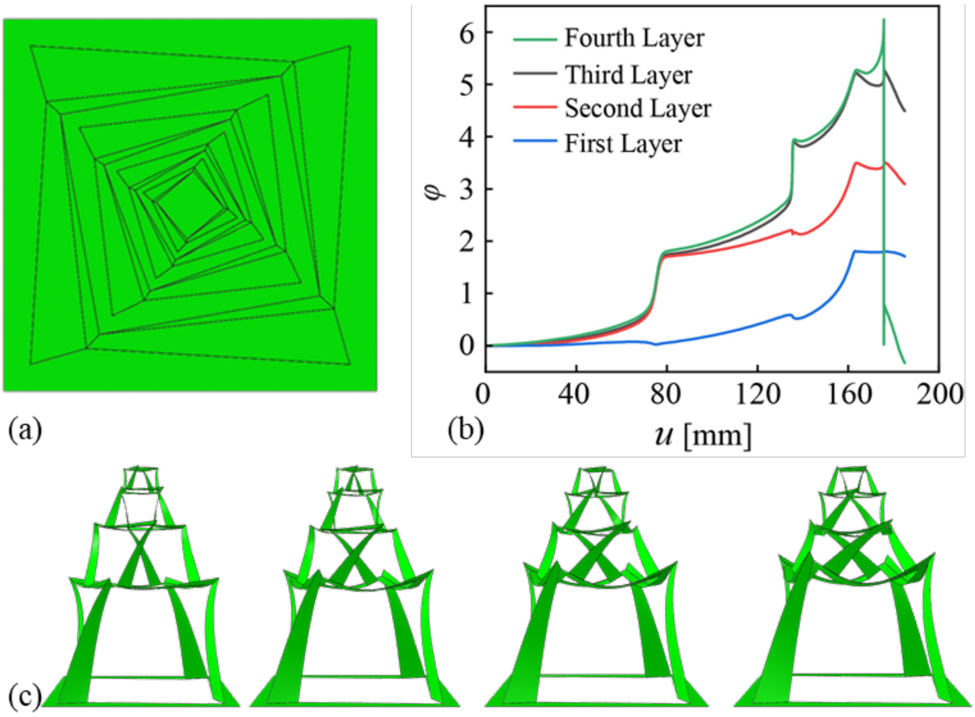

Supplementary Figure 9: Vertical four-layer array of the RFSL kirigami elements. (a) Kirigami pattern. The inner and outer layers are arranged in the same scheme, and the inner size is 0.6 of the outer layer. (b) Rotation angle of each layer. Jump information in rotation angle can reflect the forming processes of different layers. (c) self-locking behavior. The array does not transform from its three-dimensional configuration to its initial two-dimensional planar configuration when the central square hub of the fourth layer is subjected to compressive loads.

B. Programmability

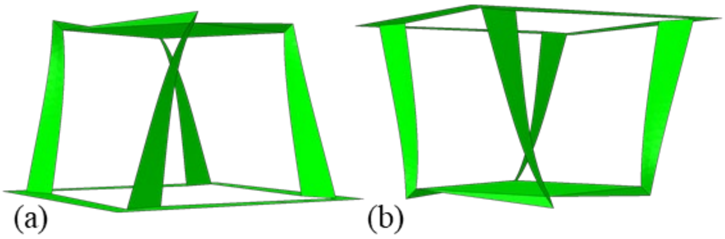

Supplementary Figure 10: Two three-dimensional stable configurations of the RFSL kirigami element. (a) "1" state and (b) "-1" state.

Supplementary Table 2. Configurations information of four-layer vertical RFSL kirigami array

No.	States	H_1 [mm]	H_2 [mm]	No.	States	H_1 [mm]	H_2 [mm]
1	1, 1, 1, 1	173.89	173.89	21	1, 0, -1, 1	79.91	68.40
2	1, 1, 1, -1	156.63	139.37	22	1, 0, -1, -1	79.91	33.88
3	1, 1, 1, 0	156.63	156.63	23	1, 0, -1, 0	79.91	51.14
4	1, 1, -1, 1	127.86	116.35	24	1, 0, 0, 1	97.17	97.17
5	1, 1, -1, -1	127.86	81.83	25	1, 0, 0, -1	79.91	62.65
6	1, 1, -1, 0	127.86	99.09	26	1, 0, 0, 0	79.91	79.91
7	1, 1, 0, 1	145.21	145.12	27	0, 1, 1, 1	93.98	93.98
8	1, 1, 0, -1	127.86	110.60	28	0, 1, 1, -1	76.72	59.46
9	1, 1, 0, 0	127.86	127.86	29	0, 1, 1, 0	76.72	76.72
10	1, -1, 1, 1	79.91	77.99	30	0, 1, -1, 1	47.95	36.44
11	1, -1, 1, -1	79.91	43.47	31	0, 1, -1, -1	47.95	1.92
12	1, -1, 1, 0	79.91	60.73	32	0, 1, -1, 0	47.95	19.18
13	1, -1, -1, 1	79.91	20.45	33	0, 1, 0, 1	65.21	65.21
14	1, -1, -1, 0	79.91	3.19	34	0, 1, 0, -1	47.95	30.69
15	1, -1, 0, 1	79.91	49.22	35	0, 1, 0, 0	47.95	47.95
16	1, -1, 0, -1	79.91	14.70	36	0, 0, 1, 1	46.03	46.03
17	1, -1, 0, 0	79.91	31.96	37	0, 0, 1, -1	28.77	11.51
18	1, 0, 1, 1	125.94	125.94	38	0, 0, 1, 0	28.77	28.77
19	1, 0, 1, -1	108.68	91.42	39	0, 0, 0, 1	17.26	17.26
20	1, 0, 1, 0	108.68	108.68				
