# Supplementary material for: Self-locking Kirigami surfaces via controlled stretching
Source: Commun Eng. 2024 Feb 7;3:26. doi: 10.1038/s44172-024-00169-5 (PMC10955878; doi:10.1038/s44172-024-00169-5)
Supplement: Supplementary file 2 — Description of Additional Supplementary Files [file 44172_2024_169_MOESM2_ESM.pdf]

Description of Additional Supplementary Files

File name: Supplementary Movie 1

Description: Active forming and self locking
